# Polymyxin Acute Kidney Injury: a case of severe tubulopathy

**DOI:** 10.1590/2175-8239-JBN-2019-0191

**Published:** 2021-04-23

**Authors:** Lucas Alexandre de Mello Goldin, Leticia Nicoletti Silva, Thiago Florencio da Silva, Vinicius Daher Alvares Delfino

**Affiliations:** 1 Hospital Evangélico de Londrina, Departamento de Nefrologia, Londrina, PR, Brasil.; 2 Pontifícia Universidade Católica, Departamento de Nefrologia, Londrina, PR, Brasil.; 3 Universidade Estadual de Londrina, Departamento de Nefrologia, Londrina, PR, Brasil.

**Keywords:** Polymyxin B, Acute Kidney Injury, Kidney Tubules, Proximal, Toxicity, Electrolytes, Polimixina B, Lesão Renal Aguda, Túbulos Renais Proximais, Toxicidade, Eletrólitos

## Abstract

Polymyxins are antibiotics developed in the 1950s. Polymyxin-induced neurotoxicity has been often described in medical literature. The same cannot be said of nephrotoxicity or tubulopathy in particular. This report describes the case of a patient prescribed polymyxin B to treat a surgical wound infection, which led to significant increases in fractional excretion of calcium, magnesium, and potassium and subsequent persistent decreases in the levels of these ions, with serious consequences for the patient. Severe hypocalcemia, hypomagnesemia, and hypokalemia may occur during treatment with polymyxin. Calcium, magnesium and potassium serum levels must be monitored during treatment to prevent life-threatening conditions.

## Resumo

Polimixinas são um grupo de antibióticos desenvolvidos na década de 1950. Seus efeitos neurotóxicos são comumente descritos na literatura, porém há menos relatos sobre seus efeitos nefrotóxicos, especialmente tubulopatias. O objetivo deste relato é descrever o uso de polimixina B em uma paciente para tratamento de infecção de ferida operatória, promovendo grande aumento das frações de excreção de cálcio, magnésio e potássio e acarretando reduções graves e persistentes desses íons, com sérias consequências para a paciente. Hipocalcemia, hipomagnesemia e hipocalemia severas podem ocorrer durante terapia com polimixina e é sugerido que sejam monitorizadas as concentrações séricas desses eletrólitos durante o tratamento como forma de evitar condições de risco à vida.

## Introduction

Polymyxins are antibiotics developed in the 1950s from *Bacillus polymyxa* strains. They work by competitively displacing calcium and magnesium ions from the cell membrane, thereby causing the cell wall of Gram-negative bacteria to rupture^1^. Polymyxins have been historically less prescribed, since other antibiotic agents with fewer side effects are available. Today, however, they are prescribed more frequently on account of increased antibiotic resistance^2^.

Side effects - neurotoxicity and nephrotoxicity in particular - have limited the use of polymyxin³. Nephrotoxicity is a multifactorial event caused in part by high levels of the drug in proximal tubule cells, increased oxidative stress, and cell death/necrosis^4^^-^ 6.

Acute Kidney Injury (AKI) is the most common clinical symptom of polymyxin-induced nephrotoxicity. A recently published review on drug-induced nephrotoxicity mentioned that hypokalemia and hypomagnesemia are often present in cases of polymyxin-induced AKI^7^.

We were unable to find reports of tubular alterations caused by polymyxin B that led to hypokalemia, hypomagnesemia, and hypocalcemia to a degree as severe as the one seen in the case described in this report. This paper reports the case of a patient prescribed polymyxin B who suffered significant tubular injury with exacerbated renal excretion of calcium, magnesium, and potassium.

## Case Report

An infectious disease physician referred a 67-year-old woman to our hospital after she failed to improve from infection in a surgical wound on her left shoulder from an orthopedic procedure, despite outpatient therapy initiated 20 days prior to her arrival at the hospital. The patient has been treated for depression with escitalopram. Her medical history included a right nephrectomy performed 20 years ago for staghorn calculi. Her serum creatinine on admission was 0.6 mg/dL.

Since the patient did not improve after initial therapy with teicoplanin for five days even with the addition of piperacillin and tazobactam, the surgical wound was debrided and specimens were sent for culturing, which came back positive for multidrug resistant *Acinetobacter baumannii* and *Pseudomonas aeruginosa*.

Treatment with polymyxin B was started with 1.5 mg/kg/day. Four days into therapy the patient complained of increased anxiety, upper extremity paresthesia, and insomnia. On the following two days, tremor in the extremities and dyspnea set in. A psychiatrist called in for a consult concluded that she was probably worsening from depression as a result of hospitalization. The patient's serum creatinine level increased from 0.6 mg/dL while she was being treated with polymyxin and plateaued at 1 mg/dL. Fifteen days into therapy with polymyxin she had dyspnea, difficulty swallowing, and muscle stiffness in association with the changes in kidney function described above, at which time the nephrology team was invited to assess the situation. Treatment with polymyxin B was discontinued.

In addition to the tests performed previously, the nephrology team ordered calcium, albumin, magnesium, venous blood gas, and urine sediment tests. The patient's total serum calcium level was 3.8 mg/dL (RR: 8.8 to 10.3 mg/dL) and her serum albumin was 2.6 mg/dL (RR: 3.5 to 5 mg/dL); her serum phosphorus level was normal (2.7 mg/dL; RR: 2.5 to 4.8 mg/dL); her serum potassium was 2.5 mEq/l (RR: 3.5 to 5 mEq/l) and her serum magnesium was 0.52 mg/dL (RR: 1.7 to 2.5 mg/dL). The patient had severe hypocalcemia, hypokalemia, and hypomagnesemia. Her venous blood gas was as follows: pH: 7.44, pCO_2_: 40.1 mmHg, HCO3^-^: 26.6 mEq/l; H^+^: 36.3 nmol/l, which was indicative of mild metabolic alkalosis. Urine sediment examination did not present signs of glucosuria or proteinuria; urine density: 1015; pH: 6.5; white cell count: 29,000; epithelial cells: 23,000; and red blood cells: 7,000/mL.

She was started on intravenous electrolyte replacement and improved markedly from symptoms within 24 hours of therapy. Serum creatinine resumed to baseline levels and the patient was administered replacement therapy with 50% magnesium sulfate (5 g/day), 10% calcium gluconate (1 g, three times a day), and 19.1% potassium chloride (50 mEq/day).

Three days into replacement therapy the patient's serum electrolyte levels had not increased substantially. Tests were ordered to verify the fractional excretion of calcium, magnesium, and potassium.

As recommended, the serum level of magnesium was multiplied by 0.7. The fractional excretion of calcium was presented solely for the purpose of observing its decrease with time and treatment, since total calcium - not ionized calcium (the fraction that actually gets filtered) - was used.

Data on the fractional excretion of calcium, magnesium, and potassium on Days 3, 5, and 7 of intravenous replacement therapy are shown in Table 1.

**Table 1:** Changes in electrolyte serum levels and fractional excretion on Days 3, 5, and 7 of parenteral replacement therapy

	Ca+^2^	Ca^+2^ u	FE Ca^+2^	Mg+2	Mg^+2^ u	FE Mg^+2^	K^+^	K^+^ u	FE K^+^
D3	7.5	16.6	13%	1.1	8.8	73%	2.5	21	54%
D5	8.3	43	9%	1.2	18	36%	4.1	54	21%
D7	8.3	13.5	5%	1.7	3.8	10%	4.07	40	33%

Key: Ca^+2^, serum calcium level, mg/dL; FE Ca^+2^, fractional excretion of calcium; Ca^+2^ u, urine calcium level, mg/dL; Mg^+2^, serum magnesium level, mg/dL; FE Mg^+2^, fractional excretion of magnesium; Mg^+2^ u, urine magnesium level, mg/dL; K^+^, serum potassium level, mEq/l; FE K^+^, fractional excretion of potassium; K^+^u, urine potassium level, mEq/l; D, day into electrolyte replacement therapy.

The patient was discharged two weeks later after discontinuation of therapy with polymyxin B. She was free from infection, her kidney function was restored to baseline levels, and she had normal serum calcium, magnesium, and potassium levels.

## Discussion

Polymyxins have been prescribed more frequently on account of the growing number of cases of infection caused by multidrug resistant Gram-negative bacteria in hospital settings. Polymyxin B and polymyxin E (colistin) have been prescribed more commonly.

Polymyxins are filtered and nearly entirely reabsorbed in the proximal convoluted tubule (PCT). A study of human tubule epithelial cells found that the intracellular concentration of polymyxin B was about 4,000 times higher than the luminal concentration of the drug.^5^ Intracellular accumulation of polymyxins in proximal tubule cells occurs via megalin-mediated endocytosis^8^.

In fact, the nephrotoxicity caused by polymyxins has been associated to cytotoxicity, more specifically in the proximal tubule cells^7^^,^ 9. Renal vasoconstriction induced by polymyxin is believed to sensitize tubule cells in areas close to the site of injury^8^.

In regard to the electrolyte level changes associated with the use of polymyxin in the case described in this report, a few comments about the management of calcium, magnesium, and potassium are warranted.

Calcium: in regard to the urinary excretion of calcium, one should realize that 50% of serum calcium is in ionized form (filtered form). Once filtered, calcium is reabsorbed along the tubules by paracellular diffusion in the PCT and in the thick ascending limb of Henle's loop (65% and 25%, respectively) and by transcellular diffusion in the distal convoluted tubules and connecting tubules (8%); in normal conditions, only about 2% of the filtered calcium is excreted in urine (fractional excretion).^10^ Our patient had total calcium measured, which did not allow the accurate estimation of her fractional excretion of calcium; however, this parameter was used to monitor her urine calcium variation with time after the introduction of calcium replacement therapy. The patient had significant urinary calcium excretion, a concerning finding in individuals with severe hypocalcemia.

Magnesium: most of the filtered magnesium is reabsorbed in the thick ascending limb of Henle's loop via permeability modulation of the tight junctions. The PCT reabsorbs 10%-20% and the distal convoluted tubule another 5% of the filtered magnesium.^11^ The kidneys respond to magnesium depletion by decreasing urinary magnesium excretion to very low levels. Therefore, daily excretion higher than 10-30 mg or fractional excretion above 2% is indicative inadequate renal loss of magnesium^12^. Our patient presented urinary levels of magnesium greater than 70% of the filtered load.

Potassium: potassium is freely filtered and almost entirely reabsorbed in the PCT and in Henle's loop. Potassium excretion varies with intake, in a response affected by factors such as circulating levels of aldosterone, urinary output, and acid-base balance. A study with 312 healthy individuals (age: 21-69 years; 213 males) reported an average fractional excretion of K+ of 8% (range: 4-16%)^13^. In cases of marked potassium depletion, urinary excretion may drop to minimal levels such as 5 to 10 mEq/day.^14^^,^ 15 In cases of significant hypokalemia, urinary potassium excretion greater than 13 mEq/day indicates the existence of a renal component in potassium loss^15^.

The changes in electrolytes along with the symptoms presented by the patient and the neurotoxic effects associated with polymyxin culminated with her referral to the intensive care unit to manage severe dyspnea.

In patients not prescribed other potentially nephrotoxic drugs, the summation of low serum calcium, magnesium, and potassium levels and elevated fractional excretion of these electrolytes might be interpreted as severe polymyxin-induced tubulopathy.

## Conclusion

Nephrotoxicity following the prescription of polymyxins is a well-known event. We were unable to find reports of significant hypocalcemia, hypomagnesemia, and hypokalemia in patients prescribed polymyxins in the literature. However, our patient presented with life-threatening electrolyte alterations that required the monitoring of serum electrolyte and creatinine levels during the course of therapy with polymyxins.
